# High-CBD Extract (CBD-X) in Asthma Management: Reducing Th2-Driven Cytokine Secretion and Neutrophil/Eosinophil Activity

**DOI:** 10.3390/ph17101382

**Published:** 2024-10-17

**Authors:** Miran Aswad, Antonina Pechkovsky, Narmeen Ghanayiem, Haya Hamza, Yaniv Dotan, Igal Louria-Hayon

**Affiliations:** 1 Medical Cannabis Research and Innovation Center, Rambam Health Care Campus, Haifa 3109601, Israel; 2 Clinical Research Institute at Rambam (CRIR), Rambam Health Care Campus, Haifa 3109601, Israel; 3 Institute of Pulmonology, Rambam Health Care Campus, Haifa 3109601, Israel

**Keywords:** CBD extracts, asthma, type 2 helper T cells (Th2), neutrophil, eosinophil, OVA-induced asthmatic mouse model, cytokines, migration, IgE

## Abstract

Background/Objectives: Asthma is a chronic inflammatory disorder of the airways affecting over 10% of the global population. It is characterized by airway inflammation, mucus hypersecretion, and bronchial hyperresponsiveness, driven predominantly by type 2 helper T cells (Th2) and type 2 innate lymphoid cells (ILC2s) in a subset of patients. However, a significant portion of asthmatic individuals present with “type 2-low” asthma that is often refractory to standard inhaled corticosteroid (ICS) therapy. Therefore, developing innovative therapeutic strategies has become essential. Recent studies have highlighted cannabidiol (CBD) as a promising anti-inflammatory agent capable of modulating immune responses. This study investigates the therapeutic potential of a high-CBD extract (CBD-X) in asthma. Methods: We evaluated the effects of CBD-X on cells involved in asthma pathogenesis using primary human Th2 cells, neutrophils, and asthma mouse model. Results: Our findings indicate that CBD-X extract inhibits Th2 differentiation and reduces the secretion of IL-5 and IL-13, which are crucial cytokines in asthma. Additionally, CBD-X significantly reduces pro-inflammatory cytokines IL-8 and IL-6 in neutrophils and impairs their migration, a critical step in airway inflammation. In a murine asthma model, CBD-X administration led to marked downregulation of IgE and pro-asthmatic cytokines, along with reduced leukocyte, eosinophil, and neutrophil infiltration in lung tissues. Conclusions: These results suggest that CBD-X extract could offer a novel and complementary approach to managing both type 2-high and type 2-low asthma by targeting key inflammatory pathways and modulating immune cell behavior.

## 1. Introduction

Asthma is a chronic inflammatory condition of the airways, characterized by coughing, wheezing, shortness of breath, and chest tightness. More than 10% of the global population is affected by this disease [1]. The pathophysiology of asthma involves airway inflammation that leads to increased mucus production [2], remodeling of the airway wall [3], and bronchial hyper-responsiveness (BHR) [4], which is the tendency of airway smooth muscle cells to react to non-specific stimuli such as allergens and cold air [5]. In many asthmatic individuals, chronic airway inflammation is primarily driven by type 2 helper T cells (Th2) or innate lymphoid cells type 2 (ILC2s) producing cytokines such as IL-4, IL-5, and IL-13 [6,7]. These type 2 cytokines are central to the disease’s hallmark features, including eosinophilia [8], IgE production [9], and increased susceptibility to exacerbations [10]. However, only approximately 50% of asthmatic patients exhibit this “type 2-high” signature. The remaining patients often present with “type 2-low” asthma, which is frequently associated with obesity, neutrophilic inflammation, and poor response to corticosteroids, the conventional treatment for asthma [11].

Inhaled corticosteroids (ICS) remain the cornerstone of therapy for persistent asthma, supplemented by inhaled short acting bronchodilators for acute relief and long acting bronchodilators for prolonged effect [12]. Despite these treatments, many patients continue to experience inadequate asthma control due to factors such as refractory disease, ineffective treatment regimens, non-adherence, and comorbid conditions [13], highlighting the urgent need to identify new and more effective treatments for asthma.

Recent interest has emerged in the potential therapeutic role of cannabis, particularly cannabidiol (CBD), in managing inflammation. Studies indicate that CBD can reduce pro-inflammatory cytokine levels and cytokine storm [14], inhibit T cell proliferation, induce T cell apoptosis [15], effect macrophages function [16,17], and decrease immune cell migration and adhesion [18]. Moreover, CBD has demonstrated anti-oxidative [19] and anti-inflammatory properties in various autoimmune conditions, including rheumatoid arthritis, allergic contact dermatitis, and multiple sclerosis [20,21,22,23].

CBD interacts with CB1 and CB2 receptors as antagonist [24], which are highly expressed in the central nervous system and immune system, but also found in other tissues [25]. Additionally, CBD modulates redox balance and inflammation through transient receptor potential (TRP) channels, PPARγ receptors, GPR receptors, 5-HT1A receptors, and adenosine A2A receptors [20,26].

CBD administration has demonstrated significant potential in treating asthma. In experimental models of allergic asthma, CBD has been shown to reduce airway inflammation and fibrosis [27,28]. Additionally, CBD has been observed to lower cytokine levels in asthma models, suggesting its ability to modulate the immune response effectively [29].

Our recent findings further support this potential. High-CBD extract (CBD-X) has been found to effectively downregulate cytokine storm both systemically and locally in inflamed lungs [30].

## 2. Results

### 2.1. CBD-X Extract Impairs Differentiation of Human CD4 T Cells into Th2 Cells and Downregulates IL-5 and IL-13 Secretion

Based on our previous research on the effects of CBD-X extracts from the High CBD- cannabis strain, on cytokine storms and lung disease [30], we were prompted to investigate its impact on chronic lung diseases such as asthma. Th2 cells play a critical role in asthma development [31], secreting the pro-inflammatory type 2 cytokines IL-5 and IL-13 [6,7]. Moreover, Th2 cells differentiate from CD4 T cells in response to cytokines such as IL-4 [32].

We first analyzed the effect of CBD-X extract on the differentiation of CD4 T cells into Th2 cells ex vivo. Isolated CD4 T cells were treated with the high-CBD extract CBD-X. The cells were then differentiated into Th2 cells over six days. Cells were subjected to flow cytometry analysis using CD4, CCR4, and CCR6 markers. The percentage of CCR4+CCR6- in the CD4 population, termed Th2 cells, was determined.

**Figure 1:**
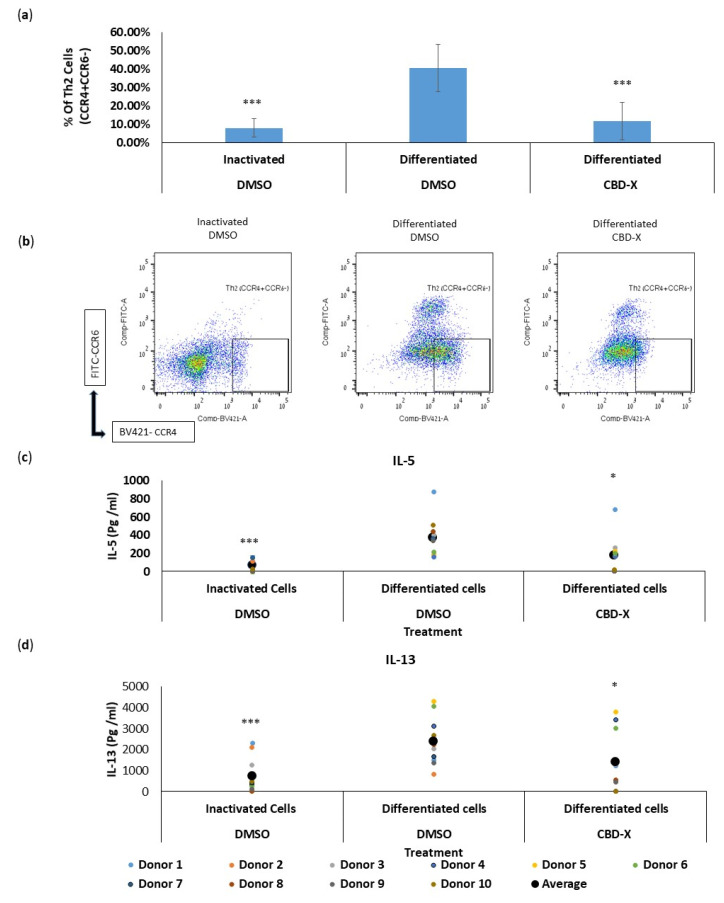
CBD-X extract attenuates the differentiation and cytokine release of human T helper 2 (Th2) cells. CBD-X extract was added to isolated differentiated CD4 T cells. Then, Th2 cells were subjected to flow cytometry analysis. CBD-X-treated cells and their control cells were stained with human APC-CD4, BV421- CCR4 and FITC-CCR6. The means and standard deviations of the percentage of Th2 (CCR4+CCR6-) from CD4 population were calculated. (**a**) The means were calculated from three different experiments of ten healthy donors. Data were analyzed in comparison to differentiated DMSO treatment representative results for CBD-X-treated cells and their control cells are shown (**b**). Moreover, supernatants were collected and levels of pro-inflammatory cytokines IL5 (**c**) and IL-13 (**d**) were detected via ELISA. The means were calculated from healthy donors (black big dot); each dot represents one case. Data were normalized to differentiated cell groups and analyzed via one-way ANOVA (Fisher’s LSD test with values *p* < 0.05 considered statistically significant, (* *p* < 0.05, *** *p* < 0.001).

Differentiated Th2 cells showed a fourfold increase in the percentage of CCR4+CCR6- cells compared to inactivated cells. However, CBD-X significantly reduced the capacity of CD4 T cells to differentiate into Th2 cells back to baseline levels (Figure 1a). The flow cytometry analysis demonstrated a marked decrease in the proportion of Th2 cells among CBD-X-treated CD4 T cells compared to the differentiated control group. Representative results of Th2 cell flow cytometry analysis are shown in three groups: inactivated, differentiated, and CBD-X differentiated cells (Figure 1b).

Given that Th2 cells secrete IL-5 and IL-13 during asthma exacerbation, we further examined the effect of CBD-X extract on the secretion of these cytokines. Differentiated Th2 cells significantly increased the secretion of IL-5 and IL-13 by threefold, compared to the control. However, CBD-X-treated Th2 cells exhibited a substantial reduction in the levels of these cytokines (Figure 1c,d).

To exclude the possibility that this observation was due to a cytotoxic effect of the added extract, cell viability was measured via Alamar Blue assay. Cell viability was not reduced with the addition of the cannabinoid samples, allowing us to conclude that the reduction in differentiation and cytokine secretion was due to a regulatory effect of CBD-X (Figure S1).

These results indicate that CBD-X extract not only impairs the differentiation process but also diminishes the functional capacity of Th2 cells, potentially impacting Th2-mediated immune responses in chronic lung diseases like asthma.

### 2.2. CBD-X Extract Attenuates the Secretion of Pro-Inflammatory Cytokines from PBMC Derived Neutrophils

**Figure 2:**
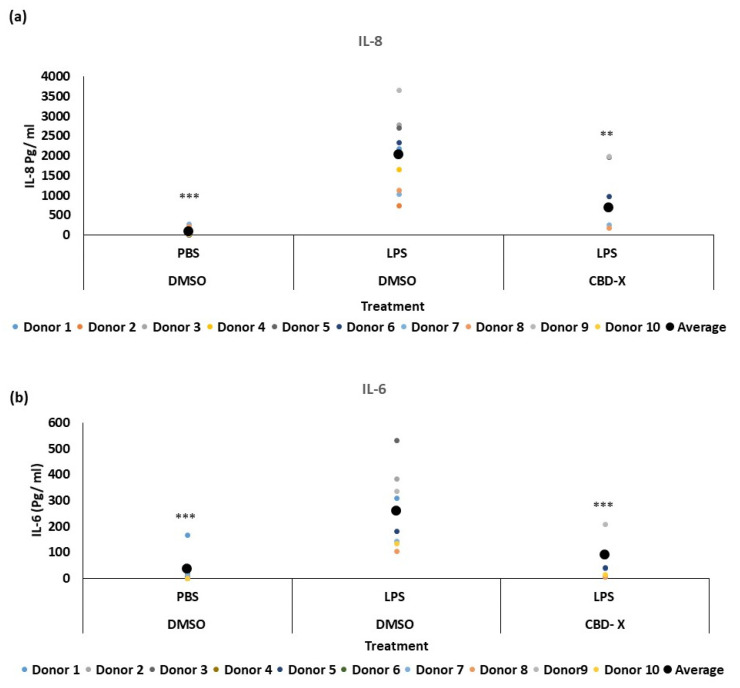
CBD-X downregulates the secretion of pro-inflammatory cytokines from human neutrophils. Isolated neutrophils were treated with 2 µg/mL CBD-X. Treated cells were activated via 100 ng/mL LPS overnight. Levels of IL-8 and IL-6 (**a**,**b**) were detected via ELISA. Each colored dot represents one donor. The means were calculated from healthy donors (black big dot) and each dot represents one case. Data were analyzed via one-way ANOVA (Fisher’s LSD test with values *p* < 0.05 considered statistically significant, (** *p* < 0.01, *** *p* < 0.001).

Some asthma patients exhibit elevated neutrophil counts in their airway secretions [33], which often show resistance to steroid treatments [34]. Neutrophilia in asthma is linked to factors such as IL-8, IL-17, and granulocyte-colony stimulating factor (G-CSF). The production of IL-8 is increased in some asthma patients [35,36], and clinical data reveal that IL-8 can serve as a marker for type 2-low asthma [37]. IL-8 is a crucial chemokine responsible for recruiting and activating neutrophils. Additionally, it is secreted by activated neutrophils, further amplifying the inflammatory response. To further investigate the role of CBD-X extract in this context, we examined its effect on neutrophils. Isolated human-derived neutrophils were pre-treated with 2 mg/mL CBD-X extract or vehicle as a control and then activated with 100 ng/mL lipopolysaccharide (LPS) overnight to induce cytokine secretion. The levels of IL-8 and IL-6 were analyzed. Our results demonstrate that LPS significantly increased the release of the pro-inflammatory cytokines IL-8 and IL-6 by about fourfold compared to the control (Figure 2a,b). However, CBD-X-treated neutrophils exhibited a significantly reduced capacity to secrete both cytokines. It is worth noting that the viability of neutrophils was not affected by the CBD-X extract at the concentration used, as determined via the Alamar Blue assay (Figure S2).

### 2.3. CBD-X Extract Inhibits IL-8 Induced Migration of Neutrophils

**Figure 3:**
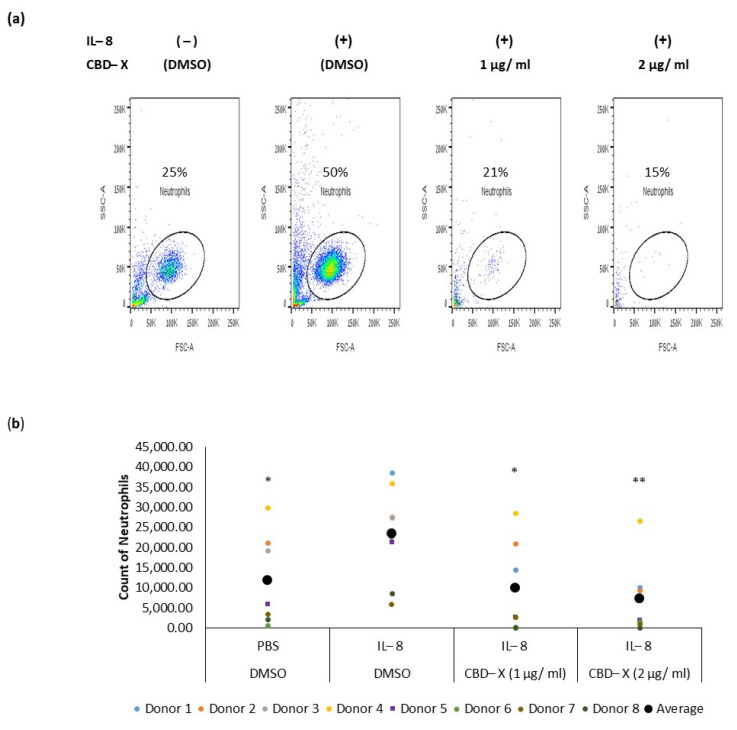
CBD-X extract inhibits the migration of neutrophils induced by IL-8. CBD-X-treated neutrophils were seeded onto the 3 µm pore polyester membrane in the upper chamber of a 24-well Boyden chamber. Human IL-8 in a concentration of 1 ng/mL was placed in the lower chamber. After three hours, cells that had migrated through the pores into the lower chamber were collected and subjected to flow cytometry analysis. (**a**) Representative results for migrated neutrophils toward IL-8 after treatment with (1 and 2 µg/mL) CBD-X. The colors in the dot plots represent the quantity of the cells. The lightest color (i.e., blue) would represent the low number of cells. In contrast, green, yellow and red would represent increasing values, with red indicating the highest values. (**b**) The number of migrated neutrophils. The means were calculated from healthy donors (black big dot) and each dot represents one case. Data were analyzed in comparison to the “IL-8, DMSO” group and analyzed via One-way ANOVA (Fisher’s LSD test with values *p* < 0.05 considered statistically significant, (* *p* < 0.05, ** *p* < 0.01).

Neutrophils are recruited to the airways of asthma patients in response to various chemokines, with IL-8 being particularly crucial for their recruitment [38,39]. IL-8 plays a significant role in asthma pathology by promoting increased neutrophilic infiltration into the lungs and exacerbating lung damage [40]. To determine whether CBD-X extract affects the migration capacity of neutrophils towards IL-8, PBMC-derived neutrophils were treated with 1 or 2 µg/mL CBD-X extract. Their ability to migrate towards IL-8 across a Boyden chamber was tested. Cells that migrated through the pores into the lower chamber were collected and subjected to flow cytometry analysis. IL-8 induced neutrophil migration significantly compared to the control. However, CBD-X extract inhibited the migration of neutrophils towards IL-8 in a dose-dependent manner (Figure 3). Neutrophil migration was inhibited by 2.5-fold and 3.5-fold with 1 and 2 µg/mL CBD-X extract, respectively (Figure 3a,b), effectively attenuating the IL-8 induced migration capacity.

These results, combined with the observed downregulation of IL-8 and IL-6 secretion by CBD-X-treated neutrophils, suggest that CBD-X may modulate the inflammatory response by impairing both neutrophil recruitment and activity. This could potentially offer a therapeutic strategy for managing conditions characterized by excessive neutrophilic inflammation, such as type 2-low asthma.

### 2.4. CBD-X Extract Modulates IgE Levels and Cytokine Secretion in a Murine Model of Asthma

**Figure 4:**
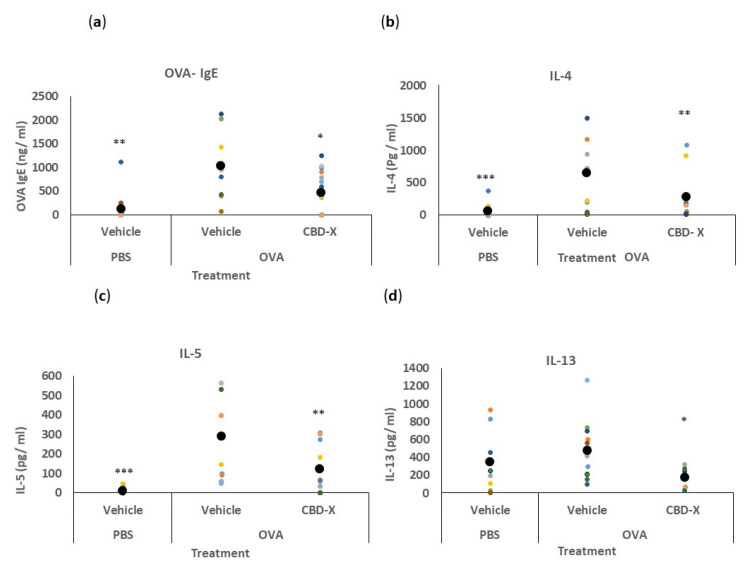
CBD-X extract attenuates serum OVA- IgE and BALF cytokine levels in an asthma mouse model. BALB/c mice were induced with OVA and were treated with 150 mg/kg CBD-X extract. Mice were euthanized, blood and lung fluids were collected. Levels of serum OVA- IgE (**a**) were detected via ELISA. Alternatively, levels of the pro-inflammatory IL-4 (**b**), IL-5 (**c**), and IL-13 (**d**) were also detected in the lung fluids via ELISA. Each colored dot represents one mouse. Standard deviations were calculated as the means of three biologically independent experiments (black big dot); *n* = 10. Data were analyzed via one-way ANOVA (Fisher’s LSD test with values *p* < 0.05 considered statistically significant, (* *p* <0.05, ** *p* < 0.01, *** *p* < 0.001).

We investigated the therapeutic potential of CBD-X extract in an in vivo mouse model of asthma. BALB/c mice were sensitized with ovalbumin (OVA, grade V) to induce an allergic response. To assess the effects of CBD-X extract, lung fluids and blood were collected and analyzed (Figure 4).

We first quantified serum IgE levels, given its role as a marker of allergic asthma [41]. The secretion of OVA-IgE levels in the serum was detected via ELISA. The increased levels of OVA-IgE in the serum of OVA-treated mice were reduced by more than twofold with the CBD-X extract (Figure 4a).

Next, we analyzed cytokine levels in the bronchoalveolar lavage fluid (BALF) from the lungs. Remarkably, the CBD-X extract led to a substantial reduction in the pro-asthmatic cytokines IL-4, IL-5, and IL-13 compared to the OVA-treated mice (Figure 4b–d).

These results indicate that CBD-X extract effectively attenuates both systemic and localized inflammatory responses associated with allergic asthma.

### 2.5. CBD-X Extract Inhibits Migration of Leukocytes to OVA-Induced Asthmatic Lungs

The asthmatic airway is characterized by chronic inflammation of the airway wall due to a complex infiltration and interplay of immune cells [11]. Therefore, we examined whether CBD-X extract affected leukocyte migration into the lungs in an OVA-treated mouse asthma model. Lung fluids were collected and immune cells were marked with anti-mouse APC-CD45, anti-mouse FITC-F4/80, anti-mouse BV786-LY6G, and PE-Siglec F for flow cytometry analysis.

**Figure 5:**
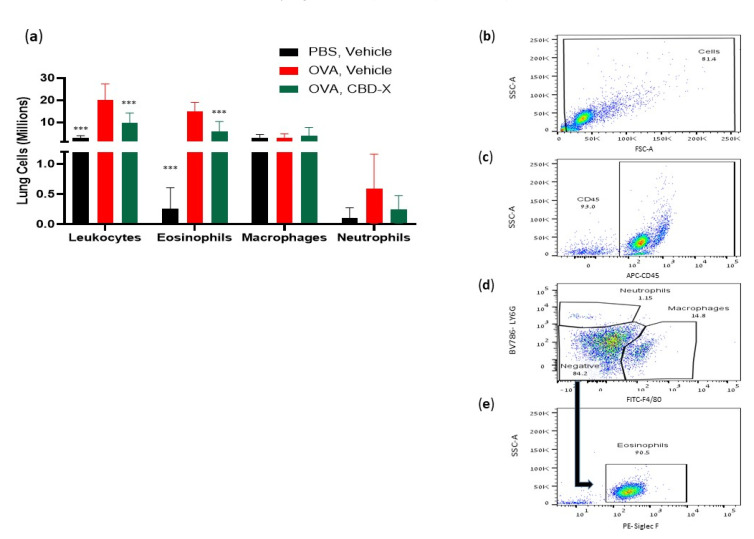
CBD-X extract inhibits migration of leukocytes to OVA-induced asthma lungs. Mice were induced with OVA-induced asthma and were treated with 150 mg/kg of CBD-X extract. Then, mice were sacrificed and lung fluids were collected. (**a**) Cells in the lung fluids were stained with anti-mouse APC-CD45, anti-mouse FITC- F480, anti-mouse BV786-LY6G, and PE- Siglec F for flow cytometry analysis. Numbers of leukocytes, eosinophils, macrophages, and neutrophils were calculated in the three different treatments; PBS- vehicle (Black bars), OVA- vehicle (Red bars) and OVA- CBD-X (green bars) (**a**). The gating strategy is (**b**) FSC vs. SSC, (**c**) APC-CD45 vs. SSC, and (**d**) BV786-LY6G vs. FITC-F4/80. (**e**) PE- Siglec F vs. SSC out of the negative population gate in (**d**). The colors in the dot plots represent the quantity of the cells. The lightest color (i.e., blue) would represent the low number of cells. In contrast, green, yellow and red would represent increasing values, with red indicating the highest values. Averages and standard deviations were calculated as the means of two biologically independent experiments, each experiment included five mice per group. Data were analyzed via mixed-effects model (Fisher’s LSD test with values *p* < 0.05 considered statistically significant, (*** *p* < 0.001).

Examination of lung immune cell populations revealed that control mice with asthma (OVA-treated mice) exhibited elevated numbers of leukocytes, and eosinophils, while neutrophil and macrophage numbers remained unchanged (Figure 5). In contrast, CBD-X-treated mice showed a significant reduction in leukocyte and eosinophil counts by 51% and 58%, respectively (Figure 5a), suggesting a targeted attenuation of the inflammatory response. Macrophage and neutrophil numbers were unaffected, indicating that CBD-X extract modulates specific immune cell populations rather than broadly suppressing immune activity.

The gating strategy of representative results is shown (Figure 5b–e). Collectively, these findings demonstrate that CBD-X treatment effectively alleviates asthma symptoms in the mouse model. The reduction in IgE levels and pro-inflammatory cytokines, along with the decreased recruitment of inflammatory cells, underscores the potential of CBD-X extract as a therapeutic agent for asthma, targeting both systemic and localized inflammatory processes.

## 3. Discussion

Asthma is a chronic inflammatory disease of the airways characterized by wheezing, shortness of breath, chest tightness, and coughing. Current treatments, such as inhaled corticosteroids (ICS), long-acting beta-agonists (LABAs), leukotriene modifiers, and biologics, aim to control symptoms and prevent exacerbations [42]. While these treatments are effective, they can have side effects ranging from oral thrush, increased risk of pneumonia, and in high doses, to systemic effects of steroids due to absorption from the lungs to the blood circuit, [43], highlighting the need for novel therapies with fewer adverse effects.

Here, we showed that the CBD-X extract of the high CBD cannabis strain, presents significant therapeutic potential in managing chronic lung diseases, particularly asthma. Our previous findings indicated that CBD-X extract had beneficial effects on cytokine storms and acute lung disease [30], which prompted us to explore its efficacy in a chronic lung disease model.

We first examined the effect of the CBD-X extract on primary human Th2 cells, as their cytokines play a central role in asthma and drive key features of the disease, including eosinophilia, mucus hypersecretion, bronchial hyper-responsiveness (BHR), IgE production, and increased susceptibility to exacerbations [11]. We observed that CBD-X extract markedly reduced the differentiation capacity of CD4 T cells into Th2 cells. This was demonstrated by a significant decrease in the proportion of Th2 cells among CBD-X-treated CD4 T cells compared to the differentiated control group. These findings suggest that CBD-X extract impaired the CD4 to Th2 differentiation process, potentially mitigating the Th2-mediated immune responses that exacerbate asthma symptoms. Th2 cells are crucial in the pathogenesis of asthma due to their secretion of IL-5 and IL-13, which promote eosinophilic inflammation and BHR.

Further analysis revealed that CBD-X-treated Th2 cells exhibited a substantial reduction in IL-5 and IL-13 secretion, underscoring the functional impairment of Th2 cells induced by CBD-X extract. Our results indicate that CBD-X not only affects the differentiation of Th2 cells but also diminishes their functional activity, thereby potentially reducing the inflammatory milieu characteristic of type 2-high asthma.

Asthma pathology is often complicated by the presence of elevated eosinophil and/or neutrophil counts in the airways, particularly in patients resistant to steroid treatments for the latter [33]. ‘Type 2-low’ asthma is more associated with obesity, presence of neutrophils, and unresponsiveness to corticosteroids [11]. Our study shows that CBD-X significantly reduces the secretion of type 2-low cytokines such as IL-8 and IL-6 from human-derived neutrophils. IL-8 is pivotal in recruiting neutrophils to the airways, thereby amplifying inflammation and lung damage [36]. CBD-X-treated neutrophils demonstrated a dose-dependent inhibition of migration towards IL-8, as shown via the Boyden chamber assay. These findings suggest that CBD-X modulates the inflammatory response by impairing neutrophil recruitment and activity, offering a novel approach to managing neutrophil-dominated inflammation in asthma. In our in vivo studies using an asthma mouse model, CBD-X treatment resulted in a notable reduction in IgE levels in the blood, a key indicator of allergic responses. Additionally, cytokine analysis of bronchoalveolar lavage fluid (BALF) from the lungs of CBD-X-treated mice revealed significant reductions in IL-4, IL-5, and IL-13 levels compared to asthmatic controls. Examination of immune cell populations in the lungs showed that control sick mice exhibited elevated numbers of leukocytes and eosinophils, but not macrophages and neutrophils. In contrast, CBD-X-treated mice demonstrated a reduction in leukocytes and eosinophils, while macrophage and neutrophil numbers remained unchanged. This selective modulation of immune cell populations by CBD-X extract highlights its potential to alleviate asthma symptoms by targeting key inflammatory pathways. Interestingly, these results align with previously reported effects of CB2 antagonists on eosinophil counts [44] and cytokine secretion [45]. This alignment may partially explain the effects of CBD-X, as CBD is known to act as a CB2 antagonist [24]. In most individuals, asthma is easily controlled with inhaled corticosteroids (ICS) and bronchodilators, usually combined in a single inhaler. However, many asthmatics do not respond adequately to these therapies and have persistent symptoms and/or exacerbations that require systemic steroids and/or hospitalization [46].

The acknowledgment that asthma is a heterogeneous disease has prompted pharmaceutical companies to develop new drugs to specifically target eosinophils, IL-5, IL4 receptor, and IgE involved in different types of asthma [11].

This evidence underscores the growing interest in CBD as a complementary treatment for asthma, highlighting its capability to address both inflammation and immune response in respiratory conditions.

In this study, we explored the therapeutic potential of CBD-X extract by examining its effects on cells involved in the asthmatic process. We utilized two ex vivo cell models—primary human Th2 cells and neutrophils—to evaluate the anti-inflammatory properties of CBD-X. Our findings revealed that CBD-X extract inhibits the differentiation of CD4+ cells into Th2 cells, leading to a reduction in the secretion of pro-asthmatic cytokines IL-5 and IL-13. Additionally, CBD-X extract decreases the levels of pro-inflammatory cytokines IL-8 and IL-6 in primary human neutrophils and impairs their migration towards IL-8. This reduction impairs the communication between immune cells, which is crucial in the development and exacerbation of asthma. Moreover, in a murine model of asthma, CBD-X administration significantly downregulated key asthma markers such as IgE and pro-asthmatic cytokines IL-4, IL-5, and IL-13 in both blood and lung tissues, and inhibited the migration of leukocytes, eosinophils, and neutrophils to lung tissues.

These results pave the way for further clinical studies to validate the efficacy and safety of CBD-X extract as a potential treatment for asthma and other chronic inflammatory lung diseases.

## 4. Materials and Methods

### 4.1. Reagents

ELISA kits for human IL-5, IL-13, IL-8, and IL-6 or mouse IL-4, IL-5, and IL-13 were obtained from R&D System (Minneapolis, MN, USA). The Mouse Anti-OVA IgE Antibody Assay Kit was obtained from Chondrex, Inc (Woodinville, WA, USA). Human TruStain FcX™ (Fc Receptor Blocking Solution), anti-human CD3 (mouse, IgG2a, κ, clone OKT3), anti-human CD28 (mouse, IgG1, κ, clone CD28.2), human IL-2 and anti-human IFN-γ (Mouse IgG1, κ, clone B27), APC anti-human CD4, (mouse, IgG2b, κ, clone A17070D), Brilliant Violet 421 anti-human CD194 (CCR4, Mouse IgG1, κ, clone L291H4) and FITC anti-human CD196 (CCR6, Mouse IgG2b, κ, clone G034E3), TruStain FcX™ (rat anti-mouse CD16/32, IgG2a, Λ, a) and APC anti-mouse CD45 (rat, IgG2b, κ, clone 30/F11), FITC anti-mouse F4/80 recombinant (Mouse IgG1, κ, clone QA17A29), Brilliant Violet 785 anti-mouse Ly-6G (Rat IgG2a, κ, clone 1A8), and PE anti-human Siglec-8 (Mouse IgG1, κ, clone 7C9) were purchased from BioLegend (San Diego, CA, USA). The media RPMI 1640 Medium with L-Glutamine were obtained from Sartorius, Beit Haemek, Israel and the medium X-VIVO 15 with gentamicin and phenol red was obtained from Lonza (Basel, Switzerland). The Human Neutrophil Isolation Kit and the Human CD4+ T -Cell Isolation Kit were obtained from STEMCELL Technologies, Vancouver, BC, Canada. Millicell Hanging Cell Culture Inserts, PET 3 µm, and the 24-well were purchased from Merck (Rahway, NJ, USA). Lipopolysaccharide (LPS) was obtained from Santa Cruz (CA, USA). Human IL4 and IL8 were obtained from PeproTech (London, UK). Fetal bovine serum (FBS), glutamine and penicillin, and 100 U/mL streptomycin were purchased from Biological Industries, Beit Haemek (Israel). Albumin from chicken egg white and lyophilized powder (Ovalbumin) were purchased from Merck (Rahway, NJ, USA). Imject™ Alum Adjuvant was purchased from Thermo Fisher Scientific (Waltham, MA, USA).

### 4.2. Cannabis Extracts

Cannabis extracts were kindly provided by Raphael Pharmaceutical, Inc (Las Vegas, NV, USA). The strains were cultivated and grown by WOLC-Way Of Life Cannabis (Israel). A concentration of 1 µg/mL CBD-X contained 35% CBD, 0.3% THC, 0% CBN, and 0.3% CBG.

### 4.3. Human Peripheral Blood Samples

Human peripheral blood samples were provided by the Israeli National Biobank for Research (MIDGAM) at Rambam Health Care Campus. The experiments were authorized by the Helsinki Committee at Rambam Health Care Campus (Authorization No. 0442-20 RMB).

### 4.4. Mice

BALB/c mice at the age of 8 to 10 weeks were purchased from Envigo, Israel. All mice were housed at a barrier-free and specific pathogen-free animal facility at the Pre-Clinical Research Authority, Technion-Israel Institute of Technology in Haifa, Israel. All experiments were performed according to the regulations of the Inspection Committee on the Constitution of the Animal Experimentation of the Technion-Israel Institute of Technology in Haifa, Israel from which authorization for performing animal studies was approved (Authorization No. IL-1330221). Experiments conformed to the regulations in the Prevention of Cruelty to Animals Law (Experiments on Animals) 5754-1994 and the Prevention of Cruelty to Animals Rules (Experiments on Animals) 5761-2001 (correct as of 1 December 2005).

### 4.5. Isolation of Immune Cells from Blood

Peripheral blood samples of healthy volunteers were collected in EDTA-containing tubes.

#### 4.5.1. CD4 Cell Isolation

CD4+ T cells were isolated via an EasySep Direct Human CD4+ T-cell Isolation Kit according to the manufacturer’s instructions (STEMCELL Technologies). In brief, isolation cocktail (50 µL/mL) and RapidSpheres (50 µL/mL) were added to the blood samples for 5 min. Samples were topped up with the recommended medium. The sample tubes were inserted into the magnet for a further 5 min. The samples were transferred to a new tube. RapidSpheres (50 µL/mL) were once more added to the samples. These were inserted into the magnet for another 5 min. The final step was repeated to produce a clear fraction.

#### 4.5.2. Neutrophil Cell Isolation

Neutrophils were isolated from blood samples via negative magnetic selection with the EasySep Direct Human Neutrophil Isolation Kit (STEMCELL Technologies) according to the manufacturer’s instructions. Briefly, Isolation Cocktail (50 µL/mL) and RapidSpheres (50 µL/mL) were added to a whole blood sample tube for 5 min. The sample tube was topped up with recommended medium and inserted into the magnet for 10 min. Then, the sample was transferred to a new tube and RapidSpheres (50 µL/mL) were added for an additional 5 min of incubation. The sample with the RapidSpheres was inserted into the magnet for 5 min and sample was transferred to a third tube. The last step was repeated to obtain a final clear fraction.

### 4.6. Cell Culture and Treatment of the Cells with Cannabis Extracts

#### 4.6.1. Th2 Cells

Firstly, 0.2 × 10^6^ Isolated CD4+ T cells were treated with 2 µg/mL cannabis extract or DMSO as a control for two hours. CD4+ T cells were cultured in RPMI 1640 or neutrophils in X-VIVO 15. After incubation, CBD-X-treated cells and control cells were centrifuged and activated or left untreated. CD4+ T cells were differentiated by seeding them on a plate coated with 2.5 µg/mL anti-human CD3 to which were added 2.5 µg/mL anti-CD28, 125 units/mL hIL-2, 20 ng/mL hIL-4, and 1 µg/mL anti-human IFN-γ in complete RPMI 1640 medium for six days. Cells were refreshed with complete RPMI 1640 medium which included hIL-2, hIL-4, and anti-human IFN-γ every three days. Then, cells were centrifuged, supernatants were collected, and levels of IL-5 and Il-13 were detected via enzyme-linked immunosorbent assay (ELISA) according to the manufacturer’s instructions (R&D System).

#### 4.6.2. Neutrophil Cells

Firstly, 0.2 × 10^6^ Isolated neutrophils were treated with 2 µg/mL cannabis extract or DMSO as a control for two hours. Then, neutrophils were activated with 100 ng/mL LPS in X-VIVO 15 medium overnight. Cells were centrifuged, supernatants were collected, and levels of IL-8 and IL-6 were detected via ELISA [30].

### 4.7. Cell Viability Measurement

CBD-X-treated Th2 or neutrophils were washed, and medium was added to the cells with 10% Alamar Blue solution. As a negative control, Alamar Blue was added to the medium without cells. The cells were further incubated for another four hours at 37 °C. The absorbance of the test and control wells was read at 570 nm and 600 nm with a standard spectrophotometer [30].

### 4.8. Cell Migration

For the cell migration experiments, 3 µm 24-well Boyden chambers were used. A total of 0.25–0.5 × 10^6^ cells/mL of neutrophils were seeded in the upper chamber of the 24-well Boyden chamber in medium. In the lower chamber, 100 ng/mL of hIL-8 were placed. Increased concentrations of cannabis extract (1 and 2 μg/mL) or DMSO as a vehicle functioning as a negative control were added to the cells. Three hours later, cells that were migrated through the pores into the lower chamber were collected and counted via flow cytometry [30].

### 4.9. Asthma Mouse Model

**Figure 6:**
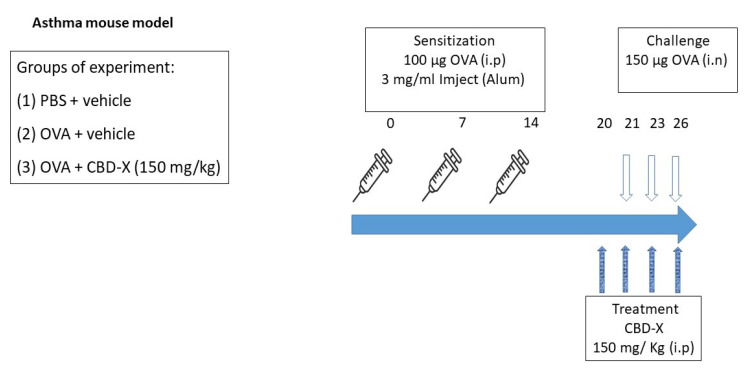
CBD-X treatment in a mouse model of OVA-induced asthma. For sensitization, BALB/c mice received an i.p injection of OVA (100 µg) with 3 mg/mL Inject (Alum) (aluminum hydroxide (40 mg/mL) and magnesium hydroxide (40 mg/mL) on days 1, 7, and 14. PBS was injected as a control. The mice were challenged with an (intranasal) i.n injection of OVA (150 µg) or PBS on days 21, 23, and 26. Mice were injected intraperitoneally with 150 mg/kg CBD-X extract or vehicle as a control on days 20, 21, 23, and 26. After 3 h of the last challenge, mice were euthanized and lung fluids were collected using PBS through lavage.

BALB/c mice were intraperitoneally (i.p) injected with 100 µg albumin, chicken egg grade V (OVA), 3 mg/mL Inject Alum (aluminum hydroxide (40 mg/mL), and magnesium hydroxide (40 mg/mL) on days 1, 7, and 14 [47,48]. Then, mice were challenged intranasally (i.n) with 150 µg OVA or PBS on days 21, 23, and 26. Mice were treated intraperitoneally with 300 mg/kg CBD-X extract or vehicle (5% kolliphor, 5% ethanol, 90% of 0.9% NaCl in sterile water for injections) as a control on days 20, 21, 23, and 26 (Figure 6). After 3 h of the last challenge, the mice were euthanized. Then, lung fluids were collected using PBS through lavage and blood was drained from the heart. Samples were centrifuged and supernatants were collected and stored at −20 °C. Levels of OVA-IgE were detected in the serum and cytokines of IL-4, IL-5, and IL-13 levels were detected in the lung fluids via ELISA. Moreover, cells in the lung fluids were stained with TruStain FcX™ (anti-mouse CD16/32) and anti-mouse APC-CD45, anti-mouse FITC- F4/80, anti-mouse BV786-LY6G, and PE- Siglec F for flow cytometry analysis.

## 5. Statistical Analysis

For statistical analysis, the ex vivo experiments data were analyzed in comparison to activated treatment as a baseline. In the case of TH2 cells, significance was determined in comparison to the “Differentiated, DMSO” group. TH2 differentiation was performed for anti-CD3, anti-CD28, hIL-2, hIL-4, and anti-IFN-γ. While in neutrophils, the significance was determined in comparison to the “LPS, DMSO” group.

In the in vivo experiments, data were analyzed in comparison to OVA-induced mice as a baseline named “OVA, Vehicle” group. Means were calculated for at least three biologically independent experiments. When each experiment included three to five mice in each group. Data were analyzed via one-way ANOVA (Fisher’s LSD test with values *p* < 0.05 considered statistically significant, (* *p* <0.05, ** *p* < 0.01, *** *p* < 0.001).

## 6. Conclusions

This study reveals novel roles of high CBD (CBD-X) extract in regulating human Th2 cell differentiation and its subsequent suppression of IL-5 and IL-13 production. In addition to inhibiting cytokine secretion, CBD-X also reduces neutrophil migration to inflamed tissues, indicating its promising therapeutic potential. The ability of CBD-X to reduce key inflammatory markers of asthma—such as IgE, IL-4, IL-5, and IL-13—in a murine asthma model further supports its anti-inflammatory effects. Additionally, CBD-X modulates immune cell recruitment to the lungs, suggesting its potential in developing new cannabis-based therapies for inflammatory disorders.

These findings indicate that CBD-X extract may provide a novel, complementary approach to managing both type 2-high and type 2-low asthma by targeting key inflammatory pathways and modulating immune responses. Further research is required to explore the molecular mechanisms underlying CBD-X’s effects. Specifically, experiments will involve treating Th2 cells and neutrophils with CBD-X to evaluate downstream inflammatory pathways. Given its therapeutic potential, CBD-X will be tested in clinical trials to assess its efficacy and safety for asthma patients.

## Data Availability

The data presented in this study are available on request from the corresponding author.

## Supplementary Materials

The following supporting information can be downloaded at: https://www.mdpi.com/article/10.3390/ph17101382/s1, Figure S1: CBD-X have no cytotoxic effect on the viability of Th2 cells; Figure S2: Cannabinoids have no cytotoxic effect on the viability of human neutrophils.
