# Supplementary material for: High-CBD Extract (CBD-X) in Asthma Management: Reducing Th2-Driven Cytokine Secretion and Neutrophil/Eosinophil Activity
Source: Pharmaceuticals (Basel). 2024 Oct 17;17(10):1382. doi: 10.3390/ph17101382 (PMC11510504; doi:10.3390/ph17101382)
Supplement: Supplementary file 1 [file pharmaceuticals-17-01382-s001.zip › pharmaceuticals-3227022-supplementary.pdf]

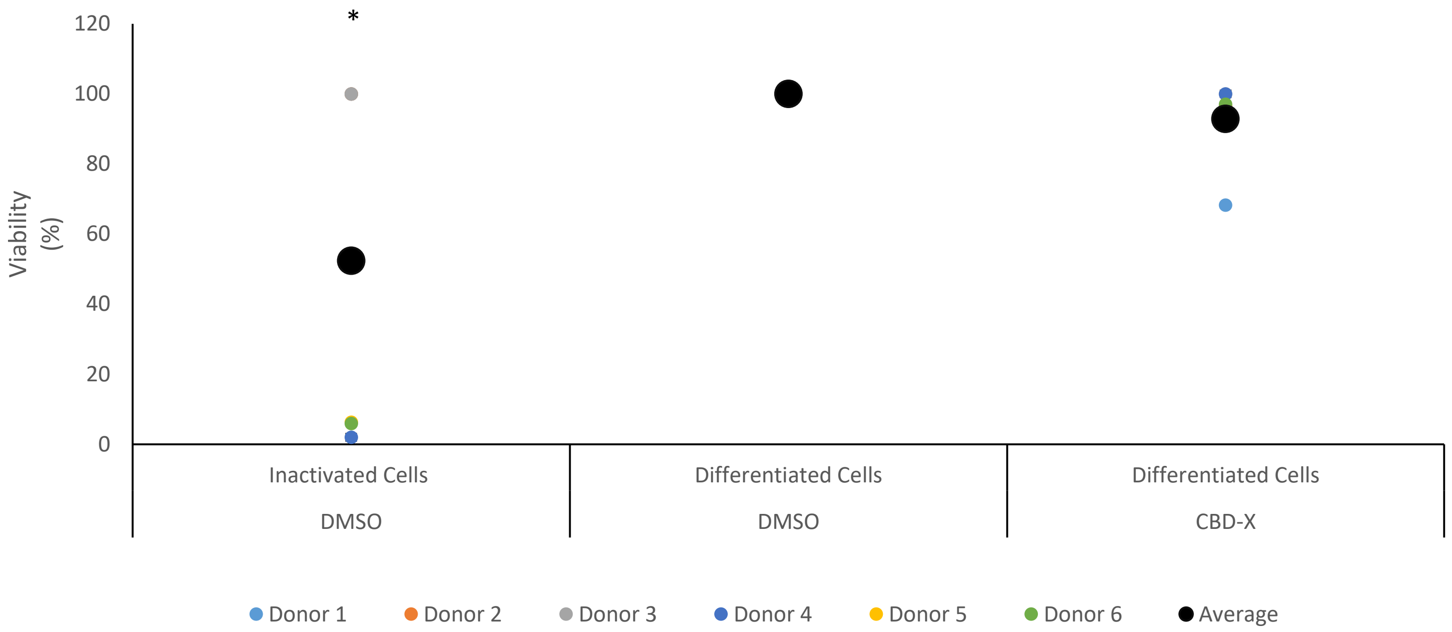

S1: CBD-X have no cytotoxic effect on the viability of Th2 cells.

The graph represents the viability of human Th2. CD4 isolated cells were left untreated (Inactivated, DMSO) or subjected to Th2 differentiation by anti-CD3, anti-CD28, h-IL-2, h-IL4 and anti IFN-γ. Differentiated Th2 cells were treated with 2 µg/ ml CBD-X(Differentiated, CBD-X) or treated with DMSO as a control (Differentiated, DMSO). The means were calculated from healthy donors (black big dot) in ratio to differentiated control and each dot represents one case. Data were analyzed by one-way ANOVA (Fisher's LSD test with values $p < 0.05$ considered statistically significant, (*p <0.05, **p < 0.01, *** p < 0.001).

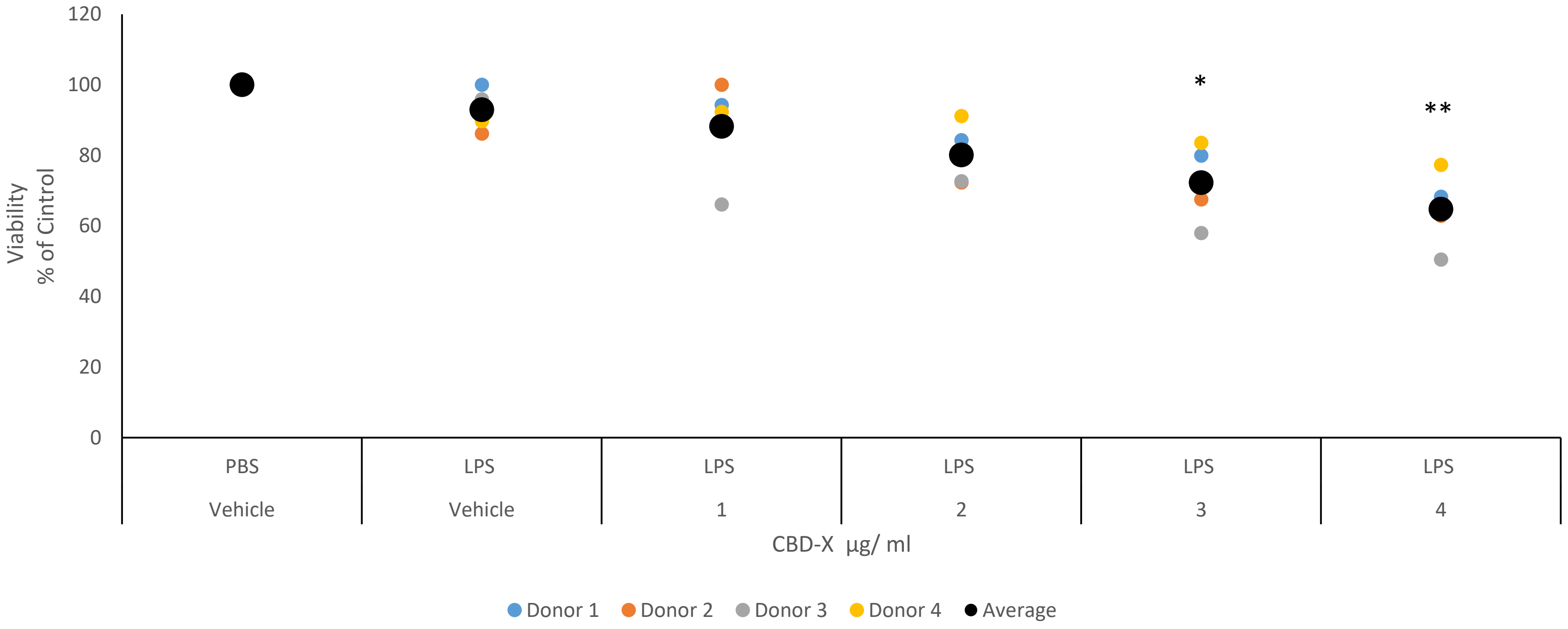

S2: Cannabinoids have no cytotoxic effect on the viability of human neutrophils. The graph represents the viability of human-derived neutrophils that were treated with 1-4 μg/ml CBD-X for 2 hours. Then, neutrophils were treated with LPS for overnight, or PBS as a control. The means were calculated from healthy donors (black big dot) in ratio to vehicle treated cells and each dot represents one case. Data were analyzed by one-way ANOVA (Fisher's LSD test with values $p < 0.05$ considered statistically significant, (*$p < 0.05$, **$p < 0.01$, *** $p < 0.001$).
